# 2518: Development of a clinically relevant rabbit surgical model for investigation of the host response to polypropylene mesh for pelvic organ prolapse

**DOI:** 10.1017/cts.2017.237

**Published:** 2018-05-10

**Authors:** Aimon Iftikhar, Bryan Brown

**Affiliations:** University of Pittsburgh, Pittsburgh, PA, USA

## Abstract

OBJECTIVES/SPECIFIC AIMS: Mesh properties, such as stiffness, porosity, and weight have been shown to correlate with the degree of mesh integration with vaginal tissue. Previous research in rhesus macaques implanted with polypropylene mesh differing in stiffness, porosity, and weight showed differences in vaginal deterioration following mesh implantation. These differences were correlated with a foreign body response, consisting primarily of activated, proinflammatory M1 macrophages. Previous studies have determined that the early macrophage polarization profile following biomaterial implantation is a strong indicator of overall tissue integration downstream. However, these early responses have not been previously observed in the appropriate surgical models. Prior work from our laboratory in developing a cytokine delivery system has shown that shifting the macrophage response at the host-implant interface from a pro-inflammatory M1 phenotype to an anti-inflammatory M2 phenotype in the first 14 days postimplantation resulted in enhanced integration of the mesh with the surrounding tissues. The present study develops an in vivo model clinically relevant surgical model to investigate the modulation of the host response to mesh. Utilizing a moderately-sized animal, we can feasibly implant mesh using the “gold standard” abdominal sacrocolpopexy procedure and evaluate the changes in the host immunologic response at early (14 d) and tissue remodeling outcomes at late stages (90 and 180 d) of implantation. METHODS/STUDY POPULATION: Commercially available heavyweight and lightweight mesh was used to investigate the modulation of the immune response. A custom MTI SILAR Automated Dip Coating machine is used to uniformly coat the mesh in a reproducible manner. An adapted radio frequency glow discharge method is used to create a stable negative charge on the surface of the mesh, followed by the sequential deposition of polycationic and polyanionic polymers to provide a stable, conformal, nanoscale coating. Chitosan served as the polycation, chosen because of its known antimicrobial and biocompatibility properties. Dermatan sulfate served as the polyanion, chosen for its important role in regulating extracellular matrix components and enhancing the activity of cytokines. Interleukin-4 (IL-4) is incorporated into the coating to be released in a controlled manner upon implantation. In vitro controlled release profiles were assessed to demonstrate efficient and local release of IL-4. Utilizing a New Zealand white rabbit surgical model, we implant mesh using the “gold standard” abdominal sacrocolpopexy procedure and evaluate the changes in the host immunologic response at early (14 d) and tissue remodeling outcomes at late stages (90 and 180 d) of implantation. The mesh-tissue complex was removed from each rabbit and processed for histological staining as well as immunolabeling of immune cells, such as macrophages. Determination of matrix metalloproteinases and fibrotic capsule formation also helps characterize the overall inflammatory response associated with each implant. RESULTS/ANTICIPATED RESULTS: We have developed a clinically relevant rabbit surgical model to implant different conditions of surgical mesh into 2 different sites, including the vagina and the abdomen. The results of this study show that implants into vaginal tissues elicited an increased host inflammatory response at 14 days as compared with those in the abdominal wall. However, at chronic time points the inflammatory response in the vagina was reduced as compared to that in the abdominal cavity. The present study also demonstrates the scale-up of a previous methodology for nano-scale coating. We present a nanometer thickness, tunable, and uniform coating capable of releasing bioactive IL-4. In vitro assays confirm the bioactivity and the controlled local release allowing for shifts in the immune response to promote implant integration. Improved remodeling has been observed to correlate with a shift in the early host response from an M1 to an M2 phenotype, however, there is limited information on the exact mechanism. Our strategy to achieve enhanced tissue remodeling demonstrate outcomes such as minimal changes to the structural properties of the mesh and a controlled release profile to sufficiently polarize macrophages around the mesh to a pro-remodeling state. DISCUSSION/SIGNIFICANCE OF IMPACT: Pelvic organ prolapse is a condition where the pelvic floor muscles weaken over time resulting in the downward shift of the pelvic organs into the vaginal canal. Moreover, factors such as obesity, age, and vaginal birth increase the susceptibility of being diagnosed with pelvic organ prolapse. Direct costs of reconstructive procedures exceed $1 billion each year in the United States. Synthetic mesh has been used to repair abdominal hernias for over half a century. Biomedical companies, through 510k and the 1976 Medical Device Amendments Act, were able to resell their hernia repair mesh as a treatment for pelvic organ prolapse. However, women who have had vaginal mesh implants have reported an increasing number of complications including chronic pain and mesh erosion/exposure at rates as high as 10%–20%. In fact, in 2008 and 2011, the US Food and Drug Administration issued warnings to doctors and patients about the mesh. In January 2016, the FDA officially had to reclassify surgical mesh for transvaginal repair of pelvic organ prolapse from a class II, moderate risk device, to a class III, high-risk device. Presently, data for the use of synthetic mesh has largely derived from abdominal hernia repair, instead of vaginal repair of prolapse. In the rodent model, the vagina is too small to implant mesh in an analogous manner to human implantation. Instead, implantations are done in the abdomen, a different tissue composition and host response profile than the vagina. Primate models of pelvic organ prolapse have been utilized, but are associated with high costs and investigation of acute immune responses are not considered ethical due to the short time of survival. Thus, our presented work will not only show the development of an improved material for implantation, but also the development of an in vivo model clinically relevant to understanding the early host response to mesh.

